# Myocardial infarction or myocarditis? A case report and review of a myocardial adverse event associated with mRNA vaccine

**DOI:** 10.3389/fmed.2023.1071239

**Published:** 2023-02-02

**Authors:** Roberto Badaró, Gustavo Novaes, Ana Cristina Andrade, Cesar Augusto de Araujo Neto, Bruna Aparecida Machado, Josiane Dantas Viana Barbosa, Milena Botelho Pereira Soares

**Affiliations:** ^1^SENAI Institute of Innovation (ISI) in Health Advanced Systems, University Center SENAI/CIMATEC, Salvador, Brazil; ^2^Allmed Specialized Clinic, Salvador, Brazil; ^3^UNIMED Hospital, Feira de Santana, Brazil; ^4^Image Diagnosis, Salvador, Brazil; ^5^Gonçalo Moniz Institute, Oswaldo Cruz Foundation (FIOCRUZ), Salvador, Brazil

**Keywords:** COVID-19, mRNA vaccine, cardiotoxicity, cardiac disease, adverse event

## Abstract

A 23-year-old man started with chest pain 8 h after his first Pfizer-BioNTech COVID-19 vaccination. ECG evaluation showed sinus tachycardia with ST-segment elevation in D1, AVL, V5, and V6, the findings compatible with acute subepicardial myocardial damage. However, cardiac MRI documented myocardial fibrosis, with cardiac late enhancement non-ischemic pattern with diffuse edema. He had no other symptoms to suggest another etiology than the vaccination. The patient was hospitalized and received corticosteroid (prednisolone) daily. Then, 2 weeks after hospitalization, all laboratory parameters and ECG were normal and the patient was discharged from the hospital. The patient had a history of Wolf-Parkinson White that was corrected with ablation when he was 11 years old. This report calls attention to myocardial adverse reaction risk for mRNA COVID-19 vaccines for people with a previous cardiac disease history.

## Introduction

Heart inflammation, such as endocarditis, myocarditis, and pericarditis, is the adverse reaction associated with mRNA vaccination reported in several countries during the COVID-19 vaccination development and after the onset of the vaccination campaign (1–6). Overall, six cases of myocarditis after the BNT162b2 vaccination were reported by Abu Mouch and Roguin et al. (1), with five patients presenting myocarditis after the second and one after the first dose of the vaccine. The six patients were men, with a median age of 23 years. Additional cases of myocarditis were also reported in individuals who received the Moderna mRNA COVID-19 vaccine (1). In adolescents and young adults, the reports of myocarditis and pericarditis were higher in frequency after the second dose than the first dose of one mRNA COVID-19 vaccine (Pfizer-BioNTech or Moderna) (1, 4, 6).

Overall, a review by the Centers for Disease Control and Prevention (CDC) of the Vaccine Adverse Events Reporting System (VAERS) identified a total of 1,226 cases of myocarditis as of 11 June 2021 (4). From 29 December 2020 to 11 June 2021, about 296 million doses of mRNA COVID-19 vaccines were administered in the USA, being 52 million to persons aged 12–29 years, receiving 30 and 22 million as first and second doses, respectively (4). The cases reported using the electronic VAERS CDC reporting system should be carefully evaluated (7), since a report to VAERS does not mean that a vaccine caused a myocardial adverse event, which should fit the diagnostic criteria for myocarditis, following the classification of the Brighton Collaboration (8). The revision of myocarditis cases reported to VAERS by the cardiologists in persons aged below 30 years reported from 1 May to 11 June 2021, and 323 cases (among 484 records) met the CDC criteria in case definitions for myocarditis, myopericarditis, or pericarditis (4).

Recently, a retrospective review of data from 5,125,696 Israeli residents which received two doses of the BNT162b2 mRNA vaccine (Pfizer-BioNTech) showed 283 having definitive or probable myocarditis attributed to the vaccination among 304 reported cases, including one fulminant fatal case (9). However, in most cases (95%—129 recipients), the myocarditis was mild, occurring in 142 persons after the first dose or a month following the second dose. In another report from Israel, the authors estimated an incidence of myocarditis of 2.13 cases/100,000 persons who had received at least one dose of vaccine [95% confidence interval (CI), 1.56–2.70], and the highest incidence (10.69 cases per 100,000 persons; 95% CI, 6.93–14.46) was reported in young male patients (aged between 16 and 29 years) (10).

The occurrence of myocarditis associated with the mRNA vaccine, on an overall risk difference between the first and second doses, has been estimated as 1.76 per 100,000 persons [95% confidence interval (CI), 1.33–2.19], with the most considerable difference among male aged between 16 and 19 years (difference, 13.73 per 100,000 persons; 95% CI, 8.11–19.46) (4, 10, 11).

In this report, we call the attention of public health services to include an advertisement for individual selection of COVID-19 type of vaccine for persons with a history of previous cardiac diseases.

## Case description

A 23-year-old man presented with oppressive chest pain that started 6–8 h after being administered with his first Pfizer-BioNTech COVID vaccination dose. His initial vital signs were a temperature of 38.7*^o^*C, blood pressure of 130/70 mmHg, heart rate of 128 bmp, and 100% oxygen saturation at the emergency room. The initial levels of cardiac enzymes evaluated were troponin *I* > 15 ng/ml (*N* = 0.04 ng/ml), CK = 1,894 U/L (*N* = 294 U/L), and CK-MB = 65.74 ng/ml (*N* = 5.0 ng/ml). Troponin I and CK-MB levels dropped to normal values 5 days later (Table 1).

**TABLE 1:** Laboratory results of cardiac enzymes.

		Date of examination					
**Examination**	**Ref. value**	**08/15/2021 10:00 PM**	**08/16/2021 02:00 AM**	**08/17/2021 05:00 AM**	**08/18/2021 05:00 AM**	**08/19/2021 06:00 AM**	**08/20/2021 06:00 AM**
CPK	294 U/L	121.25	121.25	121.25	121.25	121.25	121.26
CK-MB	0.03 ng/ml	47.73	65.76	10.14	2.30	2.08	1.17
Troponin I	0.03 ng/ml	> 15	14.42	8.24	3.54	1.08	0.18

**FIGURE 1:**
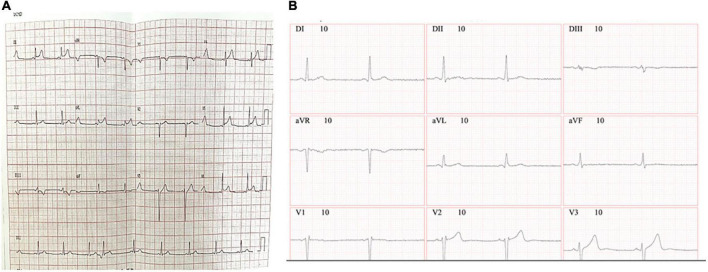
Electrocardiogram results. ECG was recorded with a set of 12-lead ECG electrodes connected to a central unit software. ECG was taken 8 h **(A)** or 13 days **(B)** after the administration of the BNT162b2 messenger RNA (mRNA) (Pfizer-BioNTech) vaccine.

The electrocardiogram (ECG) 8 h after BNT162b2 messenger RNA (mRNA) vaccine (Pfizer-BioNTech) administration on 15 August 2021 showed sinus tachycardia with ST-segment elevation in D1, AVL, V5, and V6, abnormalities compatible with acute subepicardial myocardial infarction (LV) left ventricular lateral wall (LV) (Figure 1A), whereas the ECG was normalized 13 days after vaccination (Figure 1B).

**FIGURE 2:**
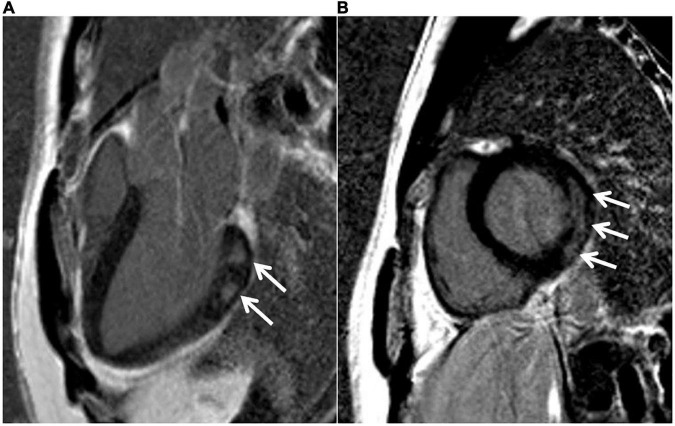
MRI results. Cardiac MRI 16 days after the administration of the BNT162b2 messenger RNA (mRNA) (Pfizer-BioNTech) vaccine, 1.5-T MR system. Delayed enhancement sequence in the long axis **(A)** and short axis **(B)**. Arrows indicate an area of myocardial fibrosis with a non-ischemic pattern in the basal lateral wall, compatible with myocarditis.

His echocardiogram with the Doppler findings at admission was a mild-to-moderate left ventricular dysfunction associated with mild pericardial effusion with a left ventricular ejection fraction of 56% and, at discharge, was 65%. Nevertheless, the cardiac magnetic resonance image (Figure 2) documented myocardial non-ischemic fibrosis 5 days later, with a cardiac enhancement non-ischemic pattern with diffuse edema. During the hospitalization, the patient received corticosteroid (prednisolone) on the following schedule: 120 mg for 3 days, 60 mg for 3 days, and 30 and 20 mg until visiting the doctor of infectious disease (ID). Then 2 weeks after hospitalization, all laboratory parameters and ECG were normal. The patient was discharged from the hospital, and during the visit to the ID doctor, the steroid therapy was suspended after performing a new ECG, which gave a normal result.

**FIGURE 3:**
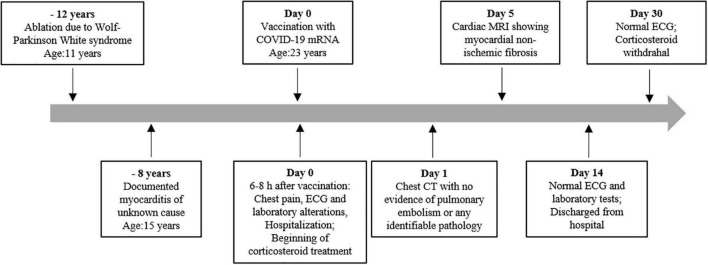
Timeline of the patient’s previous cardiac diseases, myocarditis, and pericarditis post-BNT162b2 messenger RNA (mRNA) (Pfizer-BioNTech) vaccine.

Additional evaluations by computerized tomography of the chest showed no evidence of pulmonary embolism or any identifiable pathology. The patient was not under stress, did not consume a significant amount of caffeine, and denied using any drugs or taking any over-the-counter medications (including herbals or supplements). He had no additional symptoms indicative of possible causes for the myocarditis other than the vaccine taken. He has a familiar history of Wolf-Parkinson White syndrome which was corrected with ablation when he was 11 years old. Moreover, he had a documented myocarditis of unknown cause 8 years previous to this episode. His father died of a sudden cardiac attack at the age of 50 years. A timeline of events is presented in Figure 3.

## Discussion

The SARS-CoV-2 vaccination disrupted the COVID-19 pandemic. An estimated 3.6 billion (47.6%) people in the world had received at least one dose of the COVID-19 vaccine, and the number of global COVID-19 cases and deaths dropped to less than 10% in developed countries, whereas overall, at least 40% of the population were vaccinated. In contrast, only 2.5% of people in low-income countries received at least one dose of vaccine (12). Unquestionable is the benefit of COVID-19 vaccination. By 9 October 2021, the world data reported that SARS-CoV-2S-based mRNA vaccine types were administered to 233.39 million people for the Pfizer-BioNTech and 152.84 million for the Moderna vaccine. This type of vaccine is indicated for frail patients, such as those with autoimmune diseases, transplant recipients, and patients living with HIV since they showed higher efficacy and higher estimated effectiveness (13–15). Thus, knowing predisposition factors that could determine the development of severe cardiac adverse events upon mRNA vaccination is of great relevance for vaccination programs.

Overall, solicited systemic adverse events were reported during the trials for dose definitions of the Moderna vaccine (16). The adverse reaction to this mRNA vaccine, such as local adverse events, which were nearly all mild or moderate, and pain at the injection site, was most frequently reported with a higher dose and, more frequently, after the second dose. Solicited systemic and local adverse events occurred in most adults and adolescents across both vaccinations with the Pfizer-BioNTech mRNA vaccine, including high fever, fatigue, chills, headache, myalgia, and pain at the injection site (16, 17). Among the recipients of the Pfizer-BioNTech, four serious adverse events related to the vaccine were reported (shoulder injury related to vaccine administration, right axillary lymphadenopathy, paroxysmal ventricular arrhythmia, and right leg paresthesia). Myocarditis is unquestionably a serious adverse reaction and, following mRNA vaccination, it is estimated to occur, following a second dose, at a rate of 12.6–24 cases per million, more often in adolescents and young adults at the age of 18–29 years (10, 11).

In total, two persons vaccinated with BNT162b2 died in the phase 2/3 study, one due to arteriosclerosis, and the other, by cardiac arrest (18). In another study, histopathological analysis in heart samples obtained from an autopsy of 25 individuals who were vaccinated with the mRNA vaccine for COVID-19 showed the presence of acute myocarditis with focal inflammatory foci in four persons without any signs of other possible causes of death (19). Previous cardiac diseases associated with cardiovascular adverse events were not related to the recipient’s age of the mRNA vaccine. Our patient is a young adult with previous history of cardiac arrhythmia and presented a myocarditis simulating myocardial infarction event. His ventricular dysfunction, cardiac enzyme elevations, and ECG suggested acute subepicardial myocardial infarction, which the cardiac MRI could not confirm. However, transient myocardial non-ischemia fibrosis without necrotic consequences could be shown by cardiac MRI. Moreover, myopericarditis can be associated with atrioventricular (AV) dissociation, a condition of desynchrony of electrical activity between atria and ventricles associated with ST elevation in the anterior, leading to the altered ECG and raised troponin I levels (20, 21). One limitation of this case investigation was the lack of coronary angiography, which could help to rule out myocardial infarction.

The presence of histological changes compatible with myopericarditis was shown in an experimental study after the first priming dose by intravenous route in mice. The myopericarditis persisted for 2 weeks and was aggravated by a second booster dose by intramuscular or intravenous routes (22). In addition, the gene expression of pro-inflammatory cytokines such as IL-1β, IFN-β, IL-6, and TNF-α in the hearts was significantly increased after the mRNA vaccine injection by intravenous route. Lipid nanoparticles are the most commonly utilized carriers for *in vivo* RNA delivery due to their ability to protect mRNA molecules from degradation, bring mRNA to the negatively charged cell membranes, and mediate endocytosis and endosomal escape (23, 24).

Indeed, there is no doubt that mRNA vaccines affect myocardial cells, possibly by inducing and increase in IL-18 levels (25). The CDC surveillance committee for COVID-19 vaccine adverse reactions previously recommended a precaution for the administered mRNA-based vaccine (7). Table 2 summarizes the studies describing the cardiotoxicity of SARS-CoV-2 mRNA vaccination. Importantly, a systematic literature review highlights that protective immune responses elicited by COVID-19 mRNA vaccines decline within 90–180 days, indicating the need to continuously boost the population (35).

**TABLE 2:** Reports on cardiotoxicity of SARS-CoV2 mRNA vaccines.

References	Vaccine	Number of cases	Manifestation	Country
Abu Mouch et al. (1)	BNT162b2	6	Myocarditis established by cardiac MRI	Israel
D’Angelo et al. (2)	BNT162b2	1	Myopericarditis by laboratory and cardiac MRI	Italy
Deb et al. (3)	mRNA-1273	1	Myocarditis	USA
Gargano et al. (4)	BNT162b2 and mRNA-1273	323 confirmed of 484 < 30 years	Myocarditis, pericarditis, myopericarditis confirmed by revision of cases	USA
Khogali and Abdelrahman (5)	mRNA-1273	1	Acute perimyocarditis	Qatar
Mevorach et al. (9)	BNT162b2	136	Myocarditis (definitive or probable)	Israel
Witberg et al. (10)	BNT162b2	54	Myocarditis	Israel
Larson et al. (26)	BNT162b2 and mRNA-1273	8	Myocarditis by laboratory and cardiac MRI	USA and Italy
Marshall et al. (27)	BNT162b2	7	Acute myocarditis or myopericarditis	USA
Verma et al. (28)	BNT162b2 and mRNA-1273	2	Myocarditis	USA
Freise et al. (29)	BNT162b2 and mRNA-1273	8	Cardiac symptoms compatible with myocarditis	Germany
Meier et al. (30)	BNT162b2 and mRNA-1273	1	Subclinical pericarditis established by cardiac MRI	Germany
Saadi et al. (31)	BNT162b2	1	Myocarditis	Saudi Arabia
Kim et al. (32)	BNT162b2 and mRNA-1273	4	Myocarditis by laboratory and cardiac MRI	USA
Montgomery et al. (33)	BNT162b2 and mRNA-1273	21	Myocarditis by laboratory and cardiac MRI	USA
Schwab et al. (19)	BNT162b2 and mRNA-1273	4	Acute (epi-) myocarditis by histopathological analysis	Germany
Schneider et al. (34)	BNT162b2	1	Myocarditis	Germany

We conclude that, even though the mRNA COVID-19 vaccine appears to be very safe, a careful selection of the type of COVID-19 vaccine should be done for young males with a history of cardiac disease. Additionally, chest pain with ECG alteration suggests myocarditis in patients that report being vaccinated with the mRNA COVID-19 vaccine may not necessarily be cardiac ischemic disease.

## Data availability statement

The raw data supporting the conclusions of this article will be made available by the authors, without undue reservation.

## Ethics statement

The studies involving human participants were reviewed and approved by the Ethics Committee of SENAI CIMATEC. The patients/participants provided their written informed consent to participate in this study. Written informed consent was obtained from the individual(s) for the publication of any potentially identifiable images or data included in this article.

## Author contributions

RB, GN, AA, and CA conducted the diagnostics and medical assistance. RB wrote the original draft. BM, JB, and MS edited and contributed to the literature quoted in this manuscript. All authors read and approved the submitted version.

## References

[1] Abu Mouch S Roguin A Hellou E Ishai A Shoshan U Mahamid L Myocarditis following COVID-19 mRNA vaccination. *Vaccine.* (2021). 39:3790–3. 10.1016/j.vaccine.2021.05.087 34092429 PMC8162819

[2] d’Angelo T Cattafi A Carerj M Booz C Ascenti G Cicero G Myocarditis after SARS-CoV-2 vaccination: a vaccine-induced reaction? *Can J Cardiol.* (2021) 37:1665–7. 10.1016/j.cjca.2021.05.010 34118375 PMC8187737

[3] Deb A Abdelmalek J Iwuji K Nugent K. Acute myocardial injury following COVID-19 vaccination: a case report and review of current evidence from vaccine adverse events reporting system database. *J Prim Care Community Health.* (2021) 12:21501327211029230. 10.1177/21501327211029230 34219532 PMC8255555

[4] Gargano J Wallace M Hadler S Langley G Su J Oster M Use of mRNA COVID-19 vaccine after reports of myocarditis among vaccine recipients: update from the advisory committee on immunization practices – United States, June 2021. *MMWR Morb Mortal Wkly Rep.* (2021) 70:977–82. 10.15585/mmwr.mm7027e2 34237049 PMC8312754

[5] Khogali F Abdelrahman R. Unusual presentation of acute perimyocarditis following SARS-COV-2 mRNA-1237 moderna vaccination. *Cureus.* (2021) 13:e16590. 10.7759/cureus.16590 34447639 PMC8381757

[6] Rose J McCullough P. A report on myocarditis adverse events in the U.S. Vaccine Adverse Events Reporting System (VAERS) in association with COVID-19 injectable biological products. *Curr Probl Cardiol.* (2021) [Epub ahead of print]. 10.1016/j.cpcardiol.2021.101011 34601006 PMC8483988

[7] Different COVID-19 Vaccines. *Vaccine Adverse Events Reporting System (VAERS) by the Centers for Disease Control and Prevention.* Atlanta, GA: Centers for Disease Control and Prevention (2021).

[8] Brighton Collaboration. *Myocarditis/pericarditis case definition.* Atlanta, GA: The Task Force for Global Health (2021).

[9] Mevorach D Anis E Cedar N Bromberg M Haas E Nadir E Myocarditis after BNT162b2 mRNA vaccine against Covid-19 in Israel. *N Engl J Med.* (2021) 385:2140–9. 10.1056/NEJMoa2109730 34614328 PMC8531987

[10] Witberg G Barda N Hoss S Richter I Wiessman M Aviv Y Myocarditis after Covid-19 vaccination in a large health Care Organization. *N Engl J Med.* (2021) 385:2132–9. 10.1056/NEJMoa2110737 34614329 PMC8531986

[11] Shay D Shimabukuro T DeStefano F. Myocarditis occurring after immunization with mRNA-based COVID-19 vaccines. *JAMA Cardiol.* (2021) 6:1115–7. 10.1001/jamacardio.2021.2821 34185047

[12] Coronavirus Our World in data. *Covid vaccine doses administered Our World Data.* New Delhi: Linear Logistics (2021). Available online at: https://ourworldindata.org/covid-vaccinations

[13] Murdaca G Noberasco G Olobardi D Lunardi C Maule M Delfino L Current take on systemic sclerosis patients’. vaccination recommendations. *Vaccines.* (2021) 9:1426. 10.3390/vaccines9121426 34960174 PMC8708328

[14] Milano E Ricciardi A Casciaro R Pallara E De Vita E Bavaro D Immunogenicity and safety of the BNT162b2 COVID-19 mRNA vaccine in PLWH: a monocentric study in Bari. *Italy. J Med Virol.* (2022) 94:2230–6. 10.1002/jmv.27629 35106771 PMC9015486

[15] Dib M Le Corre N Ortiz C García D Ferrés M Martinez-Valdebenito C CoV-2 vaccine booster in solid organ transplant recipients previously immunised with inactivated versus mRNA vaccines: a prospective cohort study. *Lancet Reg Health Am.* (2022) 16:100371. 10.1016/j.lana.2022.100371 36185969 PMC9503242

[16] Jackson L Anderson E Rouphael N Roberts P Makhene M Coler R An mRNA Vaccine against SARS-CoV-2 – preliminary report. *N Engl J Med.* (2020) 383:1920–31. 10.1056/NEJMoa2022483 32663912 PMC7377258

[17] Frenck R Jr Klein N Kitchin N Gurtman A Absalon J Lockhart S Safety, immunogenicity, and efficacy of the BNT162b2 Covid-19 vaccine in adolescents. *N Engl J Med.* (2021) 385:239–50. 10.1056/NEJMoa2107456 34043894 PMC8174030

[18] Polack F Thomas S Kitchin N Absalon J Gurtman A Lockhart S Safety and efficacy of the BNT162b2 mRNA Covid-19 vaccine. *N Engl J Med.* (2020) 383:2603–15. 10.1056/NEJMoa2034577 33301246 PMC7745181

[19] Schwab C Domke L Hartmann L Stenzinger A Longerich T Schirmacher P. Autopsy-based histopathological characterization of myocarditis after anti-SARS-CoV-2-vaccination. *Clin Res Cardiol.* (2022) [Epub ahead of print]. 10.1007/s00392-022-02129-5 36436002 PMC9702955

[20] Rajiah P Desai M Kwon D Flamm SD. MR imaging of myocardial infarction. *Radiographics.* (2013) 33:1383–412. 10.1148/rg.335125722 24025931

[21] Ata F Chaudhry H Bilal A Lopez M. Myopericarditis presenting as acute ST-Elevation myocardial infarction with atrioventricular dissociation. *Heart Views.* (2020) 21:284–8. 33986929 10.4103/HEARTVIEWS.HEARTVIEWS_82_20 PMC8104326

[22] Li C Chen Y Zhao Y Lung D Ye Z Song W Intravenous injection of COVID-19 mRNA vaccine can induce acute myopericarditis in mouse model. *Clin Infect Dis.* (2021) 74:1933–50. 10.1093/cid/ciab707 34406358 PMC8436386

[23] Reichmuth A Oberli M Jaklenec A Langer R Blankschtein D. mRNA vaccine delivery using lipid nanoparticles. *Ther Deliv.* (2016) 7:319–34. 10.4155/tde-2016-0006 27075952 PMC5439223

[24] Whitehead K Langer R Anderson D. Knocking down barriers: advances in siRNA delivery. *Nat Rev Drug Discov.* (2009) 8:129–38. 10.1038/nrd2742 19180106 PMC7097568

[25] Won T Gilotra N Wood M Hughes D Talor M Lovell J Increased interleukin 18-dependent immune responses are associated with myopericarditis After COVID-19 mRNA vaccination. *Front Immunol.* (2022) 13:851620. 10.3389/fimmu.2022.851620 35251049 PMC8894592

[26] Larson K Ammirati E Adler ED Cooper L Jr Hong K Saponara G Myocarditis After BNT162b2 and mRNA-1273 Vaccination. *Circulation.* (2021) 144:506–8. 10.1161/CIRCULATIONAHA.121.055913 34133884 PMC8340725

[27] Marshall M Ferguson I Lewis P Jaggi P Gagliardo C Collins J Symptomatic acute myocarditis in 7 Adolescents after pfizer-BioNTech COVID-19 vaccination. *Pediatrics.* (2021) 148:e2021052478. 10.1542/peds.2021-052478 34088762

[28] Verma A Lavine K Lin C. Myocarditis after Covid-19 mRNA vaccination. *N Engl J Med.* (2021) 385:1332–4. 10.1056/NEJMc2109975 34407340 PMC8385564

[29] Freise N Kivel M Grebe O Meyer C Wafaisade B Peiper M Acute cardiac side effects after COVID-19 mRNA vaccination: a case series. *Eur J Med Res.* (2022) 27:80. 10.1186/s40001-022-00695-y 35655235 PMC9160507

[30] Meier C Korthals D Bietenbeck M Chamling B Drakos S Vehof V Serial cardiovascular magnetic resonance studies prior to and after mRNA-Based COVID-19 booster vaccination to assess booster-associated cardiac effects. *Front Cardiovasc Med.* (2022) 9:877183. 10.3389/fcvm.2022.877183 35592407 PMC9110668

[31] Saadi S Bossei A Alsulimani L. Acute myocarditis after COVID-19 vaccination. *Saudi Med J.* (2022) 43:1270–5. 10.1093/eurheartj/ehac239 36379527 PMC10043909

[32] Kim H Jenista E Wendell D Azevedo C Campbell M Darty S Patients With Acute Myocarditis Following mRNA COVID-19 Vaccination. *JAMA Cardiol.* (2021) 6:1196–201. 10.1001/jamacardio.2021.2828 34185046 PMC8243258

[33] Montgomery J Ryan M Engler R Hoffman D McClenathan B Collins L Myocarditis Following Immunization With mRNA COVID-19 Vaccines in Members of the US Military. *JAMA Cardiol.* (2021) 6:1202–6. 10.1001/jamacardio.2021.2833 34185045 PMC8243257

[34] Schneider J Sottmann L Greinacher A Hagen M Kasper H Kuhnen C Postmortem investigation of fatalities following vaccination with COVID-19 vaccines. *Int J Legal Med.* (2021) 135:2335–45. 10.1007/s00414-021-02706-9 34591186 PMC8482743

[35] Notarte K Guerrero-Arguero I Velasco J Ver A Santos de Oliveira M Catahay J Characterization of the significant decline in humoral immune response six months post-SARS-CoV-2 mRNA vaccination: a systematic review. *J Med Virol.* (2022) 94:2939–61. 10.1002/jmv.27688 35229324 PMC9088566

